# Experiences of postpartum anxiety during the COVID-19 pandemic: A mixed methods study and demographic analysis

**DOI:** 10.1371/journal.pone.0297454

**Published:** 2024-03-07

**Authors:** Simran Mamrath, Mari Greenfield, Cristina Fernandez Turienzo, Victoria Fallon, Sergio A. Silverio

**Affiliations:** 1 Department of Women & Children’s Health, School of Life Course & Population Sciences, Faculty of Life Sciences & Medicine, King’s College London, London, United Kingdom; 2 School of Health, Wellbeing and Social Care, Department of Wellbeing, Education, Languages and Social Care, The Open University, Milton Keynes, United Kingdom; 3 Department of Psychology, Institute of Population Health, Faculty of Health and Life Sciences, University of Liverpool, Liverpool, United Kingdom; 4 School of Psychology, Faculty of Health, Liverpool John Moores University, Liverpool, United Kingdom; University of Toyama: Toyama Daigaku, JAPAN

## Abstract

**Introduction:**

The first wave of the COVID-19 pandemic saw the reconfiguration of perinatal and maternity services, national lockdowns, and social distancing measures which affected the perinatal experiences of new and expectant parents. This study aimed to explore the occurrence of postpartum anxieties in people who gave birth during the pandemic.

**Methods:**

An exploratory concurrent mixed-methods design was chosen to collect and analyse the quantitative and qualitative data of an online survey during the first UK lockdown. The survey included the Postpartum Specific Anxiety Scale–Research Short Form–for use in global Crises [PSAS-RSF-C] psychometric tool, and open-ended questions in relation to changes in birth plans and feelings about those changes and giving birth in a pandemic. Differences in measured scores were analysed for the participant’s ethnicity, sexual orientation and disability using independent Student’s *t*-tests, and for age, the analysis was completed using Pearson’s correlation. Qualitative data from open-ended questions were analysed using a template analysis.

**Results:**

A total of 1,754 new and expectant parents completed the survey between 10^th^ and 24^th^ April 2020, and 381 eligible postnatal women completed the psychometric test. We found 52.5% of participants reported symptoms consistent with a diagnosis of postnatal anxiety–significantly higher than the rates usually reported. Younger women and sexual minority women were more likely to score highly on the PSAS-RSF-C than their older or heterosexual counterparts (*p*<0.001). Younger participants reported anxieties in the ‘infant safety and welfare’ category, whilst lesbian, gay, bisexual, and pansexual participants scored highly in the ‘psychosocial adjustment to motherhood’ category.

**Discussion:**

Postpartum anxiety is under-reported, and demographic differences in the rates of postpartum anxiety are under-researched. This research demonstrates for the first time a difference in postpartum anxiety rates amongst sexual minority women.

## 1. Introduction

The SARS-CoV-2, novel coronavirus or ‘COVID-19’ pandemic and national lockdowns have affected the physical, mental, and social health and wellbeing of populations around the world [1–4]. ‘Lockdowns’ (Government-mandated stay-at-home orders) were utilised to slow the spread of the virus within the UK. Pregnant people were placed on the UK Government’s list of ‘vulnerable populations’, and were recommended to adhere to more extreme social distancing measures during lockdown, such as ‘shielding’ by avoiding all in-person social contacts [5].

This reduction in social contact for pregnant people was accompanied by an immediate reconfiguration of perinatal services. The form this reconfiguration took varied across the UK by location, but overall, there was a reduction in antenatal (70%) and postnatal appointments (56%), with many of the remaining appointments moving to online or telephone consultations [6]. NHS Trusts reported modifications to intrapartum services, with 59% stating they had implemented temporal closures of midwife led units and/or suspensions of midwives attending homebirths, whilst all had introduced limits to who could have a birth partner, who that partner could be, and the number of birth partners allowed to be present for birth or the immediate postnatal period [6]. These changes were reflected in similar international reconfigurations to maternity services, which included changes in maternity workforce, telephone or online appointments replacing face to face appointments, and reduced choice of place of birth and postnatal visits and visitors [6–10]. In the early days of the pandemic, expectant parents therefore experienced rapid changes to their plans for birth, alongside fears of contracting COVID-19 for themselves and their unborn or newborn babies, at a time when their in-person access to both healthcare professionals and support networks of family and friends were reduced. These stressors have all been shown to increase the risk of maternal depression and anxiety [11].

Prior to the pandemic, up to one in five women in the UK developed mental health problems during pregnancy or in the first year after childbirth, including anxiety [12, 13]. Poor perinatal mental health has numerous negative sequalae, including difficulties in breastfeeding and bonding [14], negative psychosocial and developmental outcomes for children [15–17], and even suicide ideation or attempts–in the UK, suicide is the leading cause of death in mothers of infants [18–20]. Postpartum anxiety levels of around 12% have been reported [21], although due to under-reporting this figure is likely to be an underestimation [22, 23]. Given the pandemic-related stressors described above, it is unsurprising that a systematic review and meta-analysis evaluating the effect of the COVID-19 pandemic on anxiety and depression of women during pregnancy and perinatal period concluded that the COVID-19 pandemic significantly increased the risk of anxiety among women during pregnancy [24].

Evidence shows that mental health problems are not experienced equally by all sections of the UK population. Experiencing racism and discrimination poses as a significant stressor that has been associated with increased mental health problems [24]. Black women, Asian women and women of multiple ethnicities have shared poor health outcomes which are relevant to the incidence of postnatal anxiety, including receiving worse pain management [25], having higher rates of maternal and neonatal mortality [26, 27], and being less likely to access mental health services [28]. Similarly, sexual minority people are more likely to experience poor mental health than heterosexual people [29]. Furthermore, when lesbian, gay, bisexual, pansexual or Queer (LGBTQ+) people access health services, research shows they frequently encounter homophobic and transphobic discrimination by healthcare staff, with consequent negative impacts upon the individual’s mental health [30]. Disabled women are also more likely than their non-disabled peers to experience perinatal mental health difficulties [31], and–along with younger mothers–to experience higher rates or postpartum anxiety [32]. This study therefore aims to explore postnatal anxiety in those who had given birth during the first UK lockdown, and to analyse these experiences by demographic categories including age, ethnicity, sexual orientation, and disability.

## 2. Methods

This study used a mixed-methods design to obtain a comprehensive understanding of anxiety amongst those who had given birth during the first UK lockdown. A global pandemic presented a unique and unexpected situation to have a baby in, therefore we aimed to explore both the content of parents worries and their levels of anxiety. Incorporating both quantitative psychometric data and qualitative data allowed for the simultaneous measurement of anxiety levels in the general sample and in subpopulations, alongside an exploration of the nature of participants’ concerns. The study followed the procedural guidelines for the design, procedure and analysis of the Good Reporting of a Mixed Methods Study (GRAMMS) [33], and reported the findings using the Standards for Reporting Qualitative Research (SRQR) [34]. Postnatal anxiety was measured using the Postpartum Specific Anxiety Scale–Research Short Form–for use in global Crisis (PSAS-RSF-C) [35], and the psychometric scores were analysed quantitatively, whilst qualitative data was gathered via open-ended questions in an online survey, and then analysed using Template Analysis [36]. The completed GRAMMS and SRQR checklists are contained at S1 and S2 Appendices respectively.

### 2.1 Design and procedure

#### 2.1.1 Study design

An online survey mixed methods survey was distributed between the 10^th^ and 24^th^ April 2020 in the UK, which comprised of three main sections; demographic questions, a psychometric test for those who had given birth, and open-ended questions which asked about the experiences of new and expectant parents. Recruitment also took place entirely online, due to the pandemic and lockdown measures. An advert for the survey was shared by various parenting groups and on social media, including Twitter, birth and parenting groups on Facebook. The two perinatal charities who were involved in designing the research, Birthrights and the Association for Improvements in Maternity Services (AIMS) also promoted the call for participants via their social media channels.

#### 2.1.2 Ethical statement and consent procedure

Those who were interested in the research clicked on a link within the advert which directed them to information about the research. If they remained interested after reading this, they then completed an online consent form which covered participation, data collection, storage and withdrawal, and gave them the option to provide details if they wished to be invited to participate in any future research. On completion of the consent form, participants answered screening questions before being directed to either the main survey or a page thanking them for their interest and explaining that they were not eligible for inclusion.

Ethical approval for the consent procedures and the study was granted by King’s College London (BDM Research Ethics Subcommittee, reference HR-19/20-18211).

#### 2.1.3 Researcher characteristics

The researchers comprise of a group of health researchers of different genders, ages, sexual orientations, and ethnicities. Some are parents, others are not. These demographic differences are reflected in the demographic questions asked and analysed. All have an interest in researching reproductive experiences and perinatal health, which is reflected in the qualitative questions asked.

### 2.2 Participants

To be eligible for inclusion, at the time of the survey participants had to either have given birth since the 9 March 2020, or be in the last trimester of pregnancy, or be the partner of someone who met those criteria. They had to be over 18, and their baby had to have been born, or be planned to be born, in the UK. People who had become parents via adoption and those who did not have access to complete the survey electronically were excluded.

The PSAS-RSF-C [35] is only for use with people who have given birth, not partners, and not people who are still pregnant. For reasons of practicability, it was therefore administered separately to those who had given birth at the time of the survey, and later to those who were pregnant when they completed the survey.

### 2.3 Measures

The PSAS-RSF-C is a 12-item research tool to measure maternal and infant focused anxieties in the first year after birth. It has been validated for use during global crises, including the COVID-19 pandemic [35]. The four factors had good reliability, with McDonald’s ω ranging from .74 to .88. The overall scale had good reliability (McDonald’s ω = .87) [35]. The tool provides an overall score of anxiety and contains four subscales: maternal competence and attachment anxieties, infant safety and welfare anxieties, practical infant care anxieties, and psychosocial adjustment to motherhood. A cut-off score of 26 out of 48 has been suggested by Silverio et al. [35]. as the optimal level at which to consider reported symptoms as consistent with a diagnosis of postnatal anxiety, though the PSAS-RSF-C remains unvalidated for clinical screening.

Each subscale in the original 51-item PSAS [37] had varying numbers of items, however, for the rapid development of the PSAS-RSF-C [35] in response to the COVID-19 health crisis, only the top three loading factors were retained for each subscale. The subscales are: Maternal Competence and Attachment Anxieties (about parent-infant relationship); Infant Safety and Welfare Anxieties (about potential harm to the baby); Practical Infant Care Anxieties (about baby’s feeding and routine); and Psychosocial Adjustment to Motherhood (about the mother or other birthing parent’s relationships and sleep).

*Post hoc* linear regression analyses examined the significant findings (age, sexual orientation) further in relation to the PSAS-RSF-C subscales.

### 2.4 Qualitative data

The open-ended questions explored how participants had first heard about COVID-19, whether the pandemic had affected their antenatal, birth or postnatal plans and experiences, and how they felt about any changes that had occurred. The questions were tailored to account for whether participants were new parents or expectant parents, and after answering the demographic questions participants were routed appropriately. A full list of the open-ended questions are available at S3 Appendix.

### 2.5 Demographics

Participants were asked whether they themselves or their partner were pregnant or had given birth, their babies’ date of birth or due date, their local healthcare Trust, their age, sexual orientation, gender, ethnicity, and if they had a disability.

### 2.6 Method of analysis

#### 2.6.1 Quantitative analysis

To examine differences in postpartum anxiety according to demographic characteristics, PSAS-RSF-C data were analysed using SPSS software. In order to perform the inferential analyses, categorical demographic variables (sexual orientation and ethnicity) were reduced to binary variables. For sexual orientation, the binary variables are 0 = heterosexual or straight participants and 1 = bisexual, lesbian, gay, Queer, and pansexual participants. For participants’ ethnicities, 0 = participants of any white ethnicity and 1 = participants of all other ethnicities, which included Black, Asian participants, and participants of multiple ethnicities.

Conflating data from people of different sexual orientations or ethnicities together is not ideal, as the experiences of people in minoritized groups may be significantly different, obscuring differences between groups. Such conflation can also contribute to ‘othering’ [38], as binary divisions can be seen as a group which is the norm, and a group which is not the norm. However, amongst the PSAS-RSF-C participants, the sample sizes of the minority groups were too small to conduct inferential analyses had they been analysed separately. Given the binary nature of the independent variables and assumed equal variance between the independent samples, we decided to use an independent Student’s *t*-test.

Furthermore, whilst the experiences of new Black mothers, new Asian mothers, and new mothers of multiple ethnicities will be different to each other, research shows that in the UK racism, both overt and covert, is a shared experience [39]. Similarly, homophobia and heterosexism are experiences shared by sexual minority groups. Therefore, PSAS-RSF-C data from ethnic minority mothers was conflated for analysis, as was data from lesbian, gay, bisexual, pansexual, and Queer mothers.

To determine which variables to include in the regression models, bivariate analyses were first conducted to assess differences in total anxiety scores according to the demographic group. Ethnicity, sexual orientation and disability were analysed using independent Student’s *t*-tests, with the demographic groups as the independent variable and the total anxiety score as the dependent variable. Age, a continuous variable, was analysed in relation to total anxiety scores using Pearson’s correlation. If the bivariate analyses were significant at p<0.05, the demographic characteristics was then included in post hoc analyses using the PSAS-RSF-C subscales. Linear regressions were conducted to evaluate whether specific domains of anxiety varied between the demographic groups.

#### 2.6.2 Qualitative analysis

Template Analysis [36, 40] was selected as the most suitable method to evaluate new and expectant parents reports of anxieties and worries about pregnancy, birth and postnatal plans or care during the first UK lockdown. Responses to the open-ended questions contained in the online survey were mapped onto a template constructed from the four sub-scales of the PSAS-RSF-C tool. Template Analysis follows a stepped procedure, which can be categorised as: (i) (re)familiarisation with the data; (ii) preliminary coding; (iii) organisation of themes; (iv) defining an initial coding template; (v) application of the initial template; (vi) finalisation of the template and application to the full dataset [40].

Transcripts were analysed using NVivo [SM], to provide a history of the development of codes and themes. The preliminary coding of the data involved assigning labels to describe the interpretation of the participants responses. The coding allowed the generation and organisation of themes, in this case utilising the four subscales of the PSAS-RSF-C namely, Maternal Competence and Attachment Anxieties, Infant Safety and Welfare Anxieties, Practical Infant Care Anxieties, and Psychosocial Adjustment to Motherhood. Any recurrent emotions or strong ideas expressed by the participants were noted. After this, a coding template was defined that captured the emotions and experiences of all the participants. The next step involved modifying, adding, or deleting the themes according to its significance in the study and finalising the template, after which the full data set was analysed according to the template.

Rigour is maintained in Template Analysis and in our study by reflexively engaging with data and analytical processes throughout all six analytical steps, as detailed above. Furthermore, accuracy checking by multiple authors [MG, SAS, CFT] in the final two stages allowed for certainty and confidence in thematic saturation (i.e. the sub-themes were well supported by data contained within the qualitative dataset).

#### 2.6.3 Triangulation

We followed a pragmatic and parallel approach to mixed-methods data triangulation. Quantitative data from the psychometric tool were analysed first, cross-tabulated with demographic data on age, ethnicity, sexual orientation and disability. This provided numerical data about the reported rates and levels of postpartum anxiety symptoms reported by different populations within the study. Where a statistically significant result was obtained, scores for that population within the subscales of the PSAS-RSF-C were examined.

The qualitative data was analysed using the four subscales of the PSAS-RSF-C to define the initial template for the Template Analysis, within which the qualitative data was organised. From this point, further iterative templates were produced from the qualitative data without reference to the quantitative data. The quantitative and qualitative findings were then brought together at the interpretation stage [41]. This approach allowed us to use the qualitative data to give depth to the findings arising from our quantitative data analysis [42].

## 3. Results

In total, 1,754 participants met the inclusion criteria, consented to be involved in the study and gave responses to the demographic questions and the open-ended questions, whilst 381 participants were eligible for and completed the PSAS-RSF-C. When the responses for the 381 participants were collated and analysed together, we found that 200 participants (52.5%) reported symptoms consistent with a diagnosis of postnatal anxiety.

### 3.1 Demographic data

In the data acquired from the full survey, shown in Table 1, over 94% (n = 1,648) of the participants who responded were white; 2.2% (n = 39) of participants had a disability; and 95.6% (n = 1,670) of respondents were heterosexual or straight, while 3.2% (n = 56) were bisexual or pansexual, 1% (n = 17) were lesbian or gay, and 0.2% (n = 4) did not disclose their sexual orientation. Also, 98.3% (n = 1,717) of the participants were cisgender women, 1.7% (n = 29) were men (including one trans man), and 0.1% (n = 1) participant was non-binary. Finally, 97.9% (n = 1,710) of the participants were the gestational parent and 2.1% (n = 37) were the non-gestational parent (see Table 1).

**Table 1:** Demographic characteristics for full survey.

	Value
Demographic characteristics	n = 1,747
Age (mean ± standard deviation)	(32.18 ± 4.32)
Ethnicity (n/%)	
White—English / Welsh / Scottish / Northern Irish / British Irish	1548 (88.6)
White–Gypsy or Irish Traveller	1(0.1)
White—Any other white background	99 (5.7)
Multiple—White and Asian	14 (0.8)
Multiple—White and Black African	2 (0.1)
Multiple—White and Black Caribbean	10 (0.6)
Multiple—Any other mixed/multiple ethnic background	20 (1.1)
Asian–Bangladeshi	1 (0.1)
Asian–Chinese	1 (0.1)
Asian–Indian	20 (1.1)
Asian–Pakistani	7 (0.4)
Asian–Any other Asian background	2 (0.1)
Black—African	6 (0.3)
Black–Caribbean	5 (0.3)
Black–Any other Black/African/Caribbean background	1 (0.1)
Other	3 (0.2)
Identity of the parents (n/%)	
Gestational parent	1,710 (97.9)
Non-gestational parent	37 (2.1)
Gender of the participants (n/%)	
Man	29 (1.7)
Woman	1,717 (98.3)
Non-binary	1 (0.1)
Gender identity–has it been the same since birth? (n/%)	
Yes	1,740 (99.6)
No	7 (0.4)
Sexual orientation (n/%)	
Heterosexual or straight	1,670 (95.6)
Lesbian or gay	17 (1.0)
Bisexual or pansexual	56 (3.2)
Other–not disclosed	4 (0.2)
Had a disability (n/%)	
Yes	39 (2.2)
No	1,708 (97.8)

The PSAS-RSF-C responses are presented in Table 2. All respondents were cisgender women and had a similar demographic profile to the wider survey sample. We found that 96.9% (n = 369) of mothers were white; 4.2% (n = 16) had a disability; and 94.8% (n = 361) of mothers were heterosexual or straight, while 3.9% (n = 15) were bisexual or pansexual and 1.3% (n = 5) were lesbian or gay.

**Table 2:** Demographic characteristics for PSAS-RSF-C participants.

For PSAS-RSF-C questionnaire: (n = 381)	
Demographic characteristics	Value
Age (mean ± standard deviation)	(33.15 ± 4.45)
Ethnicity (n/%)	
White—English / Welsh / Scottish / Northern Irish / British Irish	345 (90.6)
White—Any other white background	24 (6.3)
Multiple—White and Black Caribbean	1 (0.3)
Multiple—White and Asian	2 (0.5)
Multiple—Any other mixed/multiple ethnic background	3 (0.8)
Asian—Pakistani	1 (0.3)
Asian–Indian	5 (1.3)
Black–Caribbean	1 (0.3)
Black–African	1 (0.3)
Gender of the participants (n/%)	
Man	0 (0)
Woman	381 (100)
Non-binary	0 (0)
Gender identity–has it been the same since birth? (n/%)	
Yes	381 (100)
No	0 (0)
Sexual orientation (n/%)	
Heterosexual or straight	361 (94.8)
Lesbian or gay	5 (1.3)
Bisexual or pansexual	15 (3.9)
Had a disability (n/%)	
Yes	16 (4.2)
No	365 (95.8)

*Note*: n = number of responses given by participants for each question, % = % of total number of participants.

From the surveys and after cleaning the data of incomplete responses, the age range of participants who completed the PSAS-RSF-C were between 19 and 51 years (n = 381), whereas for those who answered the full survey, the age range was between 18 and 57 years (n = 1,747).

#### 3.1.1 Anxiety and sexual orientation

There was a statistically significant difference in the postnatal anxiety scores between heterosexual mothers (Mean = 26.1; SD = 6.57) compared to lesbian, gay, bisexual, pansexual, or Queer mothers (Mean = 31.2; SD = 8.63), t (379) = -3.26, *p* < .001.

#### 3.1.2 Anxiety and disability

**Fig 1:**
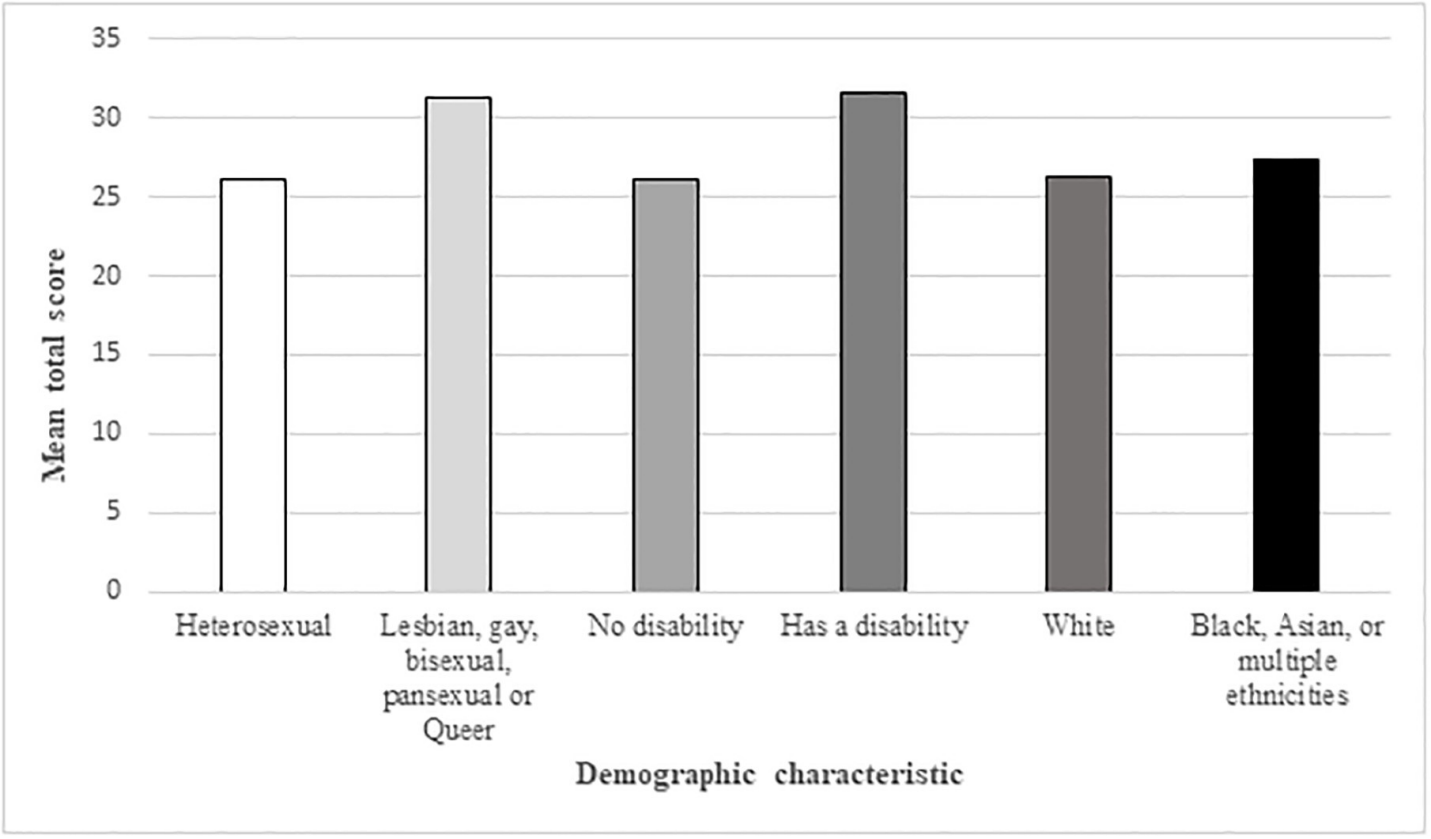
Self-reported levels of anxiety, according to ethnicity, sexual orientation and disability.

There was not a statistically significant difference in the postnatal anxiety scores between those who had a disability and those who were not disabled, t (15.5) = 2.13, *p* = 0.05. However, disabled participants average score was higher (Mean = 31.6; SD = 10.1) than those who were not disabled (Mean = 26.1; SD = 6.51). (See Fig 1).

#### 3.1.3 Anxiety and ethnicity

There was no statistically significant difference between the postnatal anxiety scores of mothers who were white, and mothers who were Black, Asian, or of multiple ethnicities, t (15.5) = 2.13, *p* = 0.05), although mothers who were Black, Asian or of multiple ethnicities had higher average scores (Mean = 27.4; SD = 7.14) than white mothers (Mean = 26.2; SD = 6.73). (See Fig 1).

#### 3.1.4 Anxiety and age

**Fig 2:**
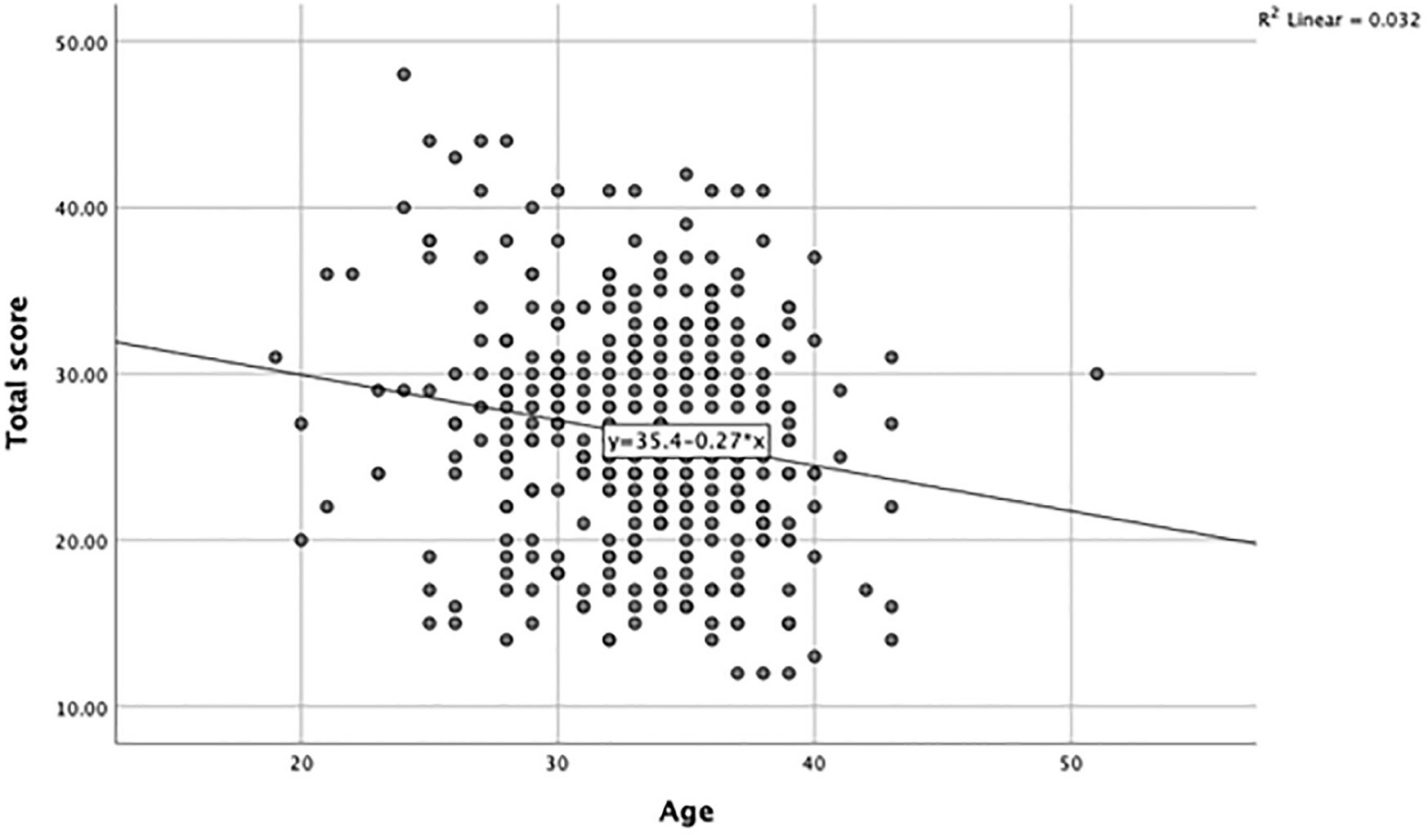
Self-reported levels of anxiety, according to age.

There was a statistically significant negative correlation between maternal age and levels of anxiety, r = -0.180, *p*<0.001 (see Fig 2).

### 3.2 PSAS-RSF-C subscale findings

Qualitative findings from the main four items of the PSAS-RSF-C scale (maternal competence and attachment anxieties; infant safety and welfare anxieties; practical infant care anxieties; psychosocial adjustment to motherhood) are presented below alongside qualitative findings for each subscale.

#### 3.2.1 Maternal competence and attachment anxieties

In this subscale, the age of the mothers was of no statistical significance (*p* = 0.494). However, we found a statistically significant relationship between the sexual orientation of the mothers and competence and attachment anxieties, with higher levels of anxiety in lesbian, gay, bisexual, pansexual and Queer mothers compared to heterosexual or straight mothers (ß = 1.320, *p* = 0.003) (Table 3).

**Table 3:** Maternal competence and attachment anxieties.

PSAS-RSF-C SUBSCALES	ß	*p*
**Maternal competence and attachment anxieties**		
Age	-0.015	0.494
Sexual orientation (heterosexual = 0; lesbian, gay, bisexual, pansexual and Queer = 1)	1.322	0.003

The final regression model predicted approximately 3% of the variance in maternal competence and attachment anxieties (R^2^ = 0.025, F (2,380) = 4.800, *p* = 0.009).

One of the recurrent themes in the qualitative responses from the participants was of the worry in mothers about the lack of support considering the announcement of lockdown and social distancing measures.

“*My mother couldn’t attend the birth*, *which she had done for my previous child*. *I had to remain in hospital with the baby for three days and my son was not allowed to visit or meet his new sister because of new restrictions*. *This was distressing for everyone*”

(Participant 12 –white, heterosexual, age 20–29 with no disability).

*“Although the midwives and doctors were wonderful and understood our situation’ it was hard to not have my husband be part of our journey*, *as it is our first baby*”

(Participant 78 –white, heterosexual, age 40–49 with no disability).

This lack of support was particularly important when it came to partners who were often excluded from antenatal care and from the early stages of labour.

*“My partner wasn’t allowed to be with me while I was induced and had to leave three hours after while I was in hospital for three further nights with no visitors”*

(Participant 39 –white and Asian, lesbian, age 20–29 and has a disability).

*“I was in hospital*, *having contractions for about 15 hours before he could come in*, *so I spent the majority of the time alone in a room… trying to rub my own back and in a lot of pain*. *I had very little support during this time”*

(Participant 180 –white, heterosexual, age 30–39 with no disability).

However, support from wider family members were also considered as important during the postpartum period. This was evident as some participants reported that due to being socially distanced from their support network during the lockdown, they were concerned about managing care of other children alongside the newborn.

*“Anxious and sad*. *Feel cheated out of newborn bonding time as the other children are around*. *Grandparents not able to meet her*. *Struggling to deal with two children and a newborn”*

(Participant 57 –white, heterosexual, age 30–39 with no disability).

#### 3.2.2 Infant safety and welfare anxieties

We found statistically significant association between both the mothers’ age as well as their sexual orientation in this subscale. Between these demographic groups, we found age to have a higher statistically significant association with anxieties in this subscale, compared to the sexual orientation of the mothers. For age, *p* = 0.001 and ß = -0.098. The negative beta value denotes younger mothers reported higher practical infant care anxiety levels than older mothers. For sexual orientation, *p* = 0.043 and ß = 1.210, again showing that lesbian, gay, bisexual and Queer mothers reported higher levels of anxiety than heterosexual mothers (see Table 4).

**Table 4:** Infant safety and welfare anxieties.

PSAS-RSF-C SUBSCALES	ß	*p*
**Infant safety and welfare anxieties**		
Age	-0.098	0.001
Sexual orientation (heterosexual = 0; lesbian, gay, bisexual, pansexual and Queer = 1)	1.208	0.043

The final regression model predicted approximately 4.1% of the variance in infant safety and welfare anxieties (R^2^ = 0.041, F (2,380) = 8.056, *p* < 0.001).

Participants expressed concerns about the effects of COVID-19 on their health as well as the health of the baby. As a result, participants reported feeling worried about the baby’s health and their own ability in taking care of the baby in the event that they themselves become unwell.

*“Anxious*! *Worried that I wouldn’t get the help I needed*, *that would be infected as would my baby”*

(Participant 18 –white, heterosexual, age 30–39 with no disability).

Changes in the provision of care and the redeployment of perinatal healthcare professionals was often reflected upon anxiously amongst participants, particularly in relation to the possibility of infection during their stay in the hospital and what this might mean for newborn babies.

*“I was just worried about cross contamination from nurses to patients”*

(Participant 270 –white, heterosexual, age 20–29 with no disability).

*“Concerned re: lack of knowledge around covid 19 effects in pregnancy and scare mongering when pregnancy women announced as at risk group despite no data to support this”*

(Participant 135 –white and Black Caribbean, heterosexual, age 30–39 with no disability).

Mothers also expressed worry about leaving their newborns in neonatal intensive care units (NICU), which is often perceived as an intimidating and stressful environment, but was made worse by the threat of COVID-19 infection.

*“I was also unable to visit our daughter regularly and required to wear a mask at all times due to limits on number of mums in the NICU room at a time*, *these restrictions have hugely affected our parent/baby bonding and been severely detrimental to my already poor mental health”*

(Participant 143 –white, heterosexual, age 30–39 and has a disability).

*“I found the unknown of it quite scary and felt and still do feel anxious about the affect it has on babies*”

Participant 136 –white, heterosexual, age 30–39 with no disability).

#### 3.2.3 Practical infant care anxieties

In this subscale, age was statistically significant, where *p* = 0.006 and ß = -0.082. The negative beta value denotes younger mothers reported higher practical infant care anxiety levels compared to older mothers. In contrast, we found no statistical significance between the sexual orientation of the mothers and this subscale, with *p* = 0.075 (see Table 5).

**Table 5:** Practical infant care anxieties.

PSAS-RSF-C SUBSCALES	ß	*p*
**Practical infant care anxieties**		
Age	-0.082	0.006
Sexual orientation (heterosexual = 0; lesbian, gay, bisexual, pansexual and Queer = 1)	1.081	0.075

The final regression model predicted approximately 3% of the variance in practical infant care anxieties (R^2^ = 0.029, F (2,380) = 5.594, *p* = 0.004).

Some of the mothers cited lack of support from the healthcare staff due to the guidelines recommending limited interactions with the patients and maintaining appropriate social distance and/or replacing face-to-face care with virtual care provisions:

“*I also didn’t get much support for breast feeding as everyone was trying to limit patient contact as a precaution*”

(Participant 127 –white, heterosexual, age 20–29 with no disability).

*“Post-natal support via phone and necessary visits has been good but wish for more breastfeeding support and have gone to external sources (NCT) for this”*

(Participant 185 –white, heterosexual, age 30–39 with no disability).

Lockdown measures were also the reason for some of the mothers reporting stress and anxiety regarding lack of support. Closure of postpartum classes and support groups consequently affected the mother’s ability to bond with their newborn and reach out to peers for practicable support.

*“It has also made things like breastfeeding harder where there are no longer any support groups”*

(Participant 137 –white, heterosexual, age 30–39 with no disability).

Lockdown measures also resulted in reduced supermarket opening hours, which in turn led to panic buying, and temporary food shortages. One participant highlighted the effect of these circumstances on her postnatally:

*“I’ve been so worried about empty shelves that I’ve hardly been eating*, *so that coupled with two hours sleep a day has impacted my energy levels and ability to breastfeed”*

(Participant 106 –white, heterosexual, age 30–39 with no disability).

#### 3.2.4 Psychosocial adjustment to motherhood

For this subscale, both age and the mother’s sexual orientation were found to be of statistical significance. Between these demographic groups, we found mothers age to have a higher statistically significant association with this subscale compared to sexual orientation of the mothers (*p* = 0.006 and *p* = 0.011 respectively). For age, we found ß = -0.069 and for sexual orientation, ß = 1.310 (see Table 6).

**Table 6:** Psychosocial adjustment to motherhood.

PSAS-RSF-C SUBSCALES	ß	*p*
**Psychosocial adjustment to motherhood**		
Age	-0.069	0.006
Sexual orientation (heterosexual = 0; lesbian, gay, bisexual, pansexual and Queer = 1)	1.305	0.011

The final regression model predicted approximately 4% of the variance in psychosocial adjustment to motherhood (R^2^ = 0.037, F (2,380) = 7.343, *p* < 0.001).

Qualitative analysis of the data highlighted a sense of isolation from the support system of the participants, who they had hoped to depend on during the postpartum period. The need for emotional and social support during the postpartum period was a recurrent theme across the responses acquired and grouped in this subscale.

*“…during my 10 day hospital stay my partner has not been allowed in to see his son or offer me any emotional support”*

(Participant 39 –white and Asian, pansexual, age 20–29 and has a disability).

One of the participants had serious concerns about her mental health and worried about developing a serious mental illness such as postpartum depression, as a result of lack of support and social isolation.

*“Most people would surround themselves with family to support both baby and new parents and obviously now that can’t happen*. *I worry lots about post-natal depression and how I will manage without that support”*

(Participant 209 –white, heterosexual, age 30–39 with no disability).

Some participants also highlighted the lack of adequate postpartum support they received from healthcare staff. They expressed feeling alone, rushed, and/or unsupported, which had an overall negative impact on their birth experience and transition to motherhood.

*“Rushed out of the post-natal ward without sufficient information regarding my section care etc*. *Cancelled home visits after discharge”*

(Participant 157 –white, heterosexual, age 20–29 with no disability).

In some instances, help and support provided by the healthcare providers proved ineffective in easing the fears and anxieties.

*“As I was in the throes of contractions*, *I found this difficult as I just needed some reassurance everything would be ok rather than being reminded of the risks although I totally appreciate why this is necessary”*

(Participant 139 –Asian-Indian, heterosexual, age 30–39 with no disability).

Mothers were also concerned about managing care of the newborn in addition to maintaining the wellbeing of other children in the family, after a difficult birth experience. Participants had expressed the need for social support to ease the transition into motherhood, which can be considered as a physically and psychologically demanding period, which was absent during the nationwide lockdown.

*“For us this means no family/friends can meet the baby*, *and we have no support in terms of toddler childcare which combined with sleepless nights makes for a challenge”*

(Participant 112– white, heterosexual, age 30–39 with no disability).

The lockdown rules announced also added complexities in organising childcare for older children during the birth of the newborn, especially since the support from family and friends was absent due to newly imposed social distancing measures.

*“It’s upsetting that friends and family are unable to support us*, *offer childcare to our eldest and meet our new baby”*

(Participant 140 –white, heterosexual, age 30–39 with no disability).

*“It also made childcare for our two year old difficult as we were in ‘lockdown’”*

(Participant 168 –white, heterosexual, age 30–39 with no disability).

## 4. Discussion

This study reports results of an online survey exploring the occurrence and experiences of postpartum specific anxieties in people who gave birth during the first UK lockdown. Overall, a high number of survey respondents reported levels of postnatal specific anxiety which were indicative of a clinical diagnosis. Our findings show that during COVID-19, different demographic groups of new parents reported different rates and symptoms of postnatal anxiety. The findings demonstrate a trend towards mothers who were either disabled or Black, Asian, or of multiple ethnicities reporting higher anxiety than their non-disabled and White counterparts respectively, but these trends were not statistically significant. Younger mothers and lesbian, gay, bisexual, pansexual or Queer mothers reported higher postnatal anxiety scores, when compared to older mothers and heterosexual mothers respectively. This finding is the first time that a correlation between sexual orientation and levels of postnatal anxiety has been identified. Results from qualitative questions provided insight into these findings, while results from the subscales showed younger mothers more likely to report infant focused postpartum specific anxiety, whereas for lesbian, gay, bisexual and pansexual mothers the anxieties were maternal focused.

It is not surprising that many women reported postnatal specific anxiety during the first UK lockdown. Numerous studies in the UK general population have identified that the whole population has experienced worsened mental health during the pandemic including higher prevalence of depressive, anxiety, and insomnia symptoms relative to pre-pandemic epidemiological data [43–45]. Young people and women have consistently been found to experience greater mental health difficulties [45]—which would align with our findings on maternal postnatal anxiety—along with parents with young children [45].

### 4.1 Increased anxiety in younger mothers

Studies examining the relationship between perinatal mental health and maternal age are conflicting in their findings; some report that younger maternal age correlates with a higher incidence of perinatal mental health difficulty such as anxiety [46, 47], whilst other literature reports a correlation between older maternal age and postnatal anxiety levels [48, 49]. Whilst our results appear to support evidence on increased postnatal anxiety in young mothers, we must acknowledge that the pandemic itself had a differential impact on people of different ages. The evidence demonstrating that young adults reported the greatest increase in psychological distress in April 2020, when compared to the results acquired in 2018 could account for some of our findings [50].

In our study, younger mothers’ anxieties were focused upon their infants, rather than anxiety for themselves or about their parenting abilities. It is possible that younger mothers may feel increased anxiety about their infants because of parity; they are more likely to be primiparous, and primiparous women tend to be more anxious about their infants than multiparous women [51]. It is also possible that other factors such as adjustment to parenthood, play a role; it brings significant changes to parents’ lives, including new parental responsibilities or worries about infant care, feeding or sleeping. These changes can be associated with negative effects in relation to a parent’s self-perceptions of competence in their role, which in turn may give rise to increased anxiety [52, 53].

### 4.2 Increased anxiety in lesbian, gay, bisexual pansexual and Queer mothers

In our study, LGBQ+ gestational mothers reported higher levels of postnatal anxiety compared to heterosexual mothers, and, in contrast to young mothers, their anxieties were maternally focused, particularly in relation to self-efficacy perceptions and worries surrounding family relationships, self-care and finances.

One of the reasons for this heightened anxiety could be the discrimination, societal stigma and unconscious bias pervasive in healthcare systems worldwide, including the NHS, against minority groups [22] including the LGBTQ+ community. Fuelling this problem is the limited research into the health of these groups, as well as the barriers they face in accessing and engaging with healthcare. The few studies which have explored sexual minority women, non-binary people and trans men’s experiences have highlighted the cisnormativity, heterosexism, intrusive questioning, and fear of or actual discrimination they experience [54–56]. Such experiences may explain the lower accessing of healthcare services by LGBQ women, and the finding from our previously published paper showing an increased consideration of freebirth as a birth option amongst LGBQ women compared to heterosexual women, during the first wave of the COVID-19 lockdown [57].

Another factor relevant to anxiety amongst postpartum LGBQ+ mothers is the invisibility of the group in the healthcare policies. When individuals experience exclusion, as well as overt or covert discrimination, there can be an amplification of the psychosocial minority stress they face which may consequently result in increased anxiety and mental health disorders [58]. The invisibility of the LGBQ+ mothers is evident in the National Maternity Review, which excluded lesbian non-birth mothers from being labelled as ‘new mothers’, due to an erroneous assumption that all new mothers will have given birth [59], as did the NHS Long Term Plan [60].

Paternal mental health has been shown to moderate the association between maternal anxiety and child outcomes [61] and it is likely that a similar mechanism operates within same-sex couples, with the non-birth mother’s mental health moderating the birthing mother’s mental health. Whilst in this study we did not measure the anxiety levels of non-birth mothers, we note that the literature shows that their experiences of discrimination and the lack of recognition as an equal parent may amplify their invisibility and contribute to anxiety [62]. One explanation of the higher levels of postpartum anxiety reported by LGBQ+ birth mothers in our study could be that a similar indirect amplification effect was in place, related to anxiety their partner experienced about exclusion or lack of recognition as a parent.

In the general population, different rates of mental health difficulties have been reported by bisexual women and lesbian women, with bisexual women experiencing higher rates of anxiety, suicidal thoughts and self-harm [30, 63]. This may be fuelled by both bi-invisibility [64, 65], and monosexist stances [66, 67]. The few studies that report on the discrete experiences of bisexual mothers have found that bisexual mothers and mothers-to-be score significantly lower on vitality, emotional, and mental health scales, and significantly higher in state anxiety scores, relative to other sexual minority mothers, and that LGBQ+ mothers who report some sexual activity with men in the past five years reported significantly higher levels of depression, state anxiety, and drug use [68]. We therefore recognise the need for further research to examine the differences in postpartum anxiety between subgroups of LGBTQ+ people.

### 4.3 Limitations

This study was conducted during the first wave of the COVID-19 pandemic, which imposed certain limitations on this research, namely that participation was only possible for those with internet access, as the recruitment and research was conducted entirely online. This means we have not gathered data from parents with reduced or no access to digital tools to complete the online questionnaires, who we anticipate might have reported heightened anxieties due to their circumstances in line with the existing literature [69]. Numbers of disabled participants were very low, and this is likely to account for the fact that no statistically significant differences in anxiety levels were found. Due to the numbers of participants in some demographic groups, we were forced to group participants in binary ways for analysis, such as heterosexual versus lesbian, gay, bisexual, pansexual and Queer; and white participants’ data versus participants who were Asian, Black, and of multiple ethnicities. Such grouping is problematic, as it may obscure differences between groups. Such grouping may also explain our finding that differences in anxiety levels between participants of different ethnicities were not statistically significant. In the main survey, there were participants who were trans men and non-binary people, however as none completed the PSAS-RSF-C, we can offer no perspective on their postnatal anxiety levels. Repeating the research with a more ethnically diverse sample, while documenting the socioeconomic power of and educational levels of the postpartum mothers in addition to the demographic variables we studied would allow for a more rigorous analysis.

### 4.4 Conclusion

To our knowledge, this was the earliest mixed methods study examining the effect of the pandemic on postnatal anxiety in the UK. The findings add to the body of evidence that rates of reported postpartum anxiety vary by the age of the birthing parent. Whilst researchers have previously postulated that LGBTQ+ parents might experience higher levels of perinatal mental health difficulties such as anxiety and depression [70, 71], we believe this is the first study worldwide to report primary data evidencing increased anxiety in postpartum LGBQ+ women. This research adds to the evidence suggesting sexual minority women accessing NHS services are concerned about or experience stigma, discrimination, and lack of support, both covert and overt. Given the lifelong consequences of impaired perinatal mental health for parents and their children, we believe it is important to ensure such services are accessible to young parents and LGBTQ+ parents.

## Supporting information

S1 Appendix Good reporting of a mixed methods study. (DOCX)

S2 Appendix Standards for reporting qualitative research. (DOCX)

S3 Appendix Full list of the open-ended questions. (DOCX)
