# Supplementary material for: Experiences of postpartum anxiety during the COVID-19 pandemic: A mixed methods study and demographic analysis
Source: PLoS One. 2024 Mar 7;19(3):e0297454. doi: 10.1371/journal.pone.0297454 (PMC10919661; doi:10.1371/journal.pone.0297454)
Supplement: S1 Appendix — (DOCX) [file pone.0297454.s001.docx]

**Appendix 1 – Good Reporting of A Mixed Methods Study (GRAMMS) checklist**

| **Guideline** | **Section: page** |
| --- | --- |
| Describe the justification for using a mixed methods approach to the research question | Methods – pg.5-6 |
| Describe the design in terms of the purpose, priority and sequence of methods | Methods – Design and procedure, pg.6 |
| Describe each method in terms of sampling, data collection and analysis | Methods – Design and procedure, Participants, Measures, Qualitative data, and Analysis pg.5-11 |
| Describe where integration has occurred, how it has occurred and who has participated in it | Methods – Qualitative analysis, Triangulation, pg.10-2 |
| Describe any limitation of one method associated with the presence of the other method | Methods – Quantitative analysis, pg.10  Discussion – Limitations, pg.33 |
| Describe any insights gained from mixing or integrating methods | Discussion - Increased anxiety in younger mothers, and Increased anxiety in lesbian, gay, bisexual pansexual and Queer mothers, pg.30-1 |
