# Supplementary material for: Experiences of postpartum anxiety during the COVID-19 pandemic: A mixed methods study and demographic analysis
Source: PLoS One. 2024 Mar 7;19(3):e0297454. doi: 10.1371/journal.pone.0297454 (PMC10919661; doi:10.1371/journal.pone.0297454)
Supplement: S2 Appendix — (DOCX) [file pone.0297454.s002.docx]

**Appendix 2 – Standards for Reporting Qualitative Research (SRQR) checklist**

**
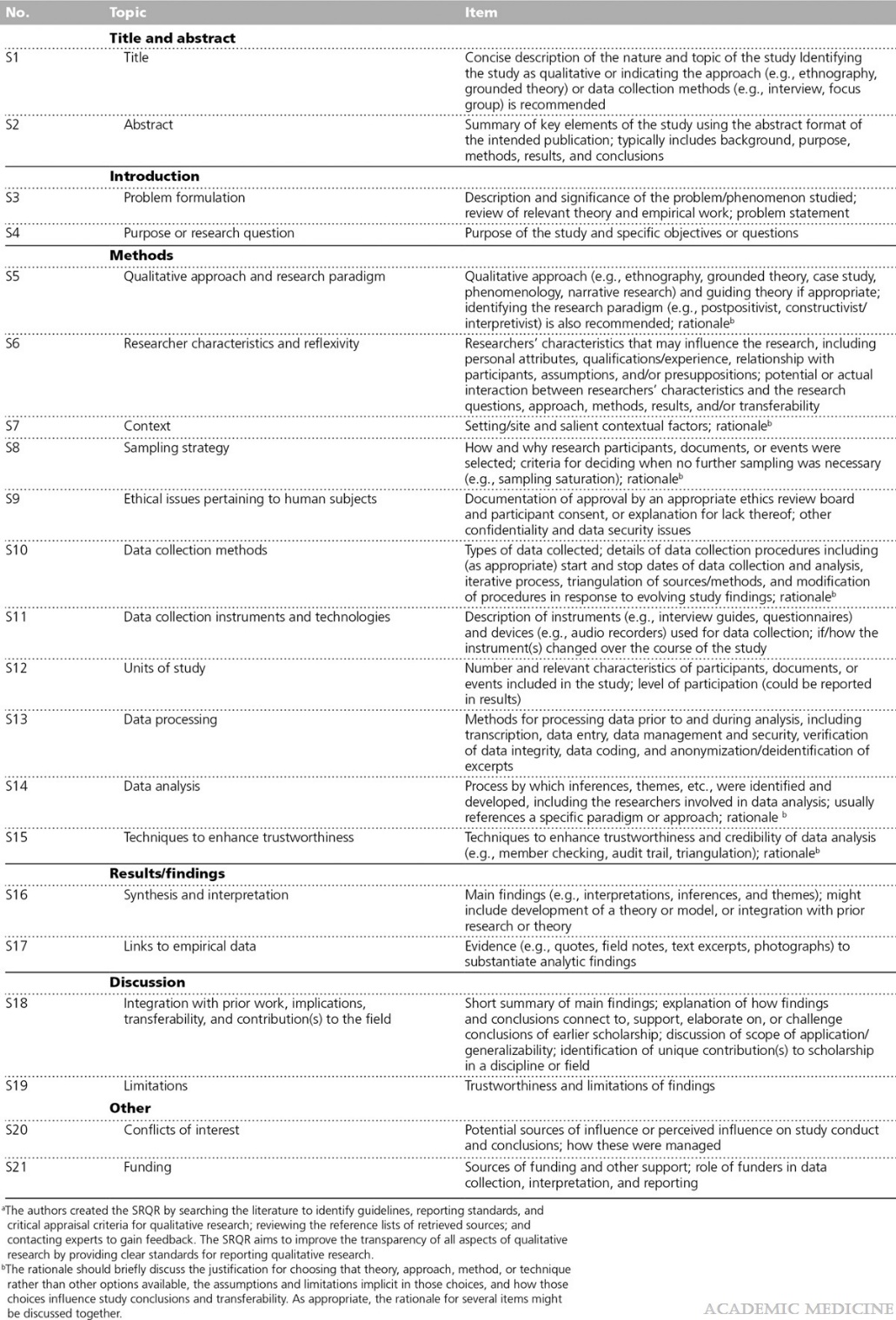
**

| **Section: page** |
| --- |
|  |
| Title – page 1 |
| Abstract – page 2 |
|  |
| Introduction – page 3 |
| Introduction – page 6 |
|  |
| Methods – page 6 |
| Methods – page 7 |
| Methods – page 7 |
| Methods – page 7 |
| Methods – page 7 |
| Methods – page 9 |
| Methods – page 8 |
| Methods – page 8 |
| Methods – page 12 |
| Methods – page 10 |
| Methods – page 11 |
|  |
| n/a – main findings are quantitative |
| Results – pages 19-27 |
|  |
| Discussion – pages 27-31 |
| Discussion – page 31 |
| n/a |
| Title page |
