# Supplementary material for: Experiences of postpartum anxiety during the COVID-19 pandemic: A mixed methods study and demographic analysis
Source: PLoS One. 2024 Mar 7;19(3):e0297454. doi: 10.1371/journal.pone.0297454 (PMC10919661; doi:10.1371/journal.pone.0297454)
Supplement: S3 Appendix — (DOCX) [file pone.0297454.s003.docx]

**Appendix 3 – Full list of the open-ended questions**

| For participants whose baby had not been born at the time of the survey |
| --- |
| What date is your baby due?  Which NHS Trust is providing your/your partner’s maternity care?  Are you/your partner under the care of an independent or private midwife or obstetrician?  Before the pandemic, what were your plans for birth?  When and how did you become aware of COVID-19?  Have your preparations for birth (such as attending antenatal classes, using a doula, having pregnancy massages) changed because of COVID-19?  How have these changes made you feel?  When and how did you become aware that COVID-19 might affect your plans for birth?  What effect has it had on your plans for birth?  How do you feel about these changes?  How do you feel about giving birth at this time when COVID-19 is prevalent in the UK? |

| For participants whose baby had been born before completion of the survey |
| --- |
| Was your baby born in the UK?  When was your baby born?  Which NHS Trust provided your partner’s maternity care?  Are you/your partner under the care of an independent or private midwife or obstetrician?  Before the pandemic, what were your plans for birth?  When and how did you become aware of COVID-19?  When and how did you become aware that COVID-19 might affect your plans for birth?  What effect did it have on your baby's birth?  How do you feel about these changes?  How do you feel about giving birth at this time when COVID-19 is prevalent in the UK? |
